# Co-Crystal of Rosiglitazone With Berberine Ameliorates Hyperglycemia and Insulin Resistance Through the PI3K/AKT/TXNIP Pathway *In Vivo* and *In Vitro*

**DOI:** 10.3389/fphar.2022.842879

**Published:** 2022-04-28

**Authors:** Qichen He, Bo Chen, Gang Wang, Duanfang Zhou, Hongfang Zeng, Xiaoli Li, Yi Song, Xiaoping Yu, Wenxin Liang, Huiling Chen, Xu Liu, Qiuya Wu, Lihong Wu, Limei Zhang, Huizhen Li, Xiangnan Hu, Weiying Zhou

**Affiliations:** ^1^ Department of Pharmacology, College of Pharmacy, Chongqing Medical University, Chongqing, China; ^2^ Chongqing Key Laboratory of Drug Metabolism, Chongqing Medical University, Chongqing, China; ^3^ Key Laboratory for Biochemistry and Molecular Pharmacology of Chongqing, Chongqing Medical University, Chongqing, China; ^4^ Department of Medicinal Chemistry, College of Pharmacy, Chongqing Medical University, Chongqing, China

**Keywords:** co-crystal, insulin resistance, type 2 diabetes mellitus, glucolipid metabolism, KKAy mice

## Abstract

**Background:** Type 2 diabetes mellitus (T2DM) is a chronic metabolic disease characterized by insulin resistance and hyperglycemia. This study examined the effect and elucidated the mechanism of improvement of hyperglycemia and insulin resistance by a co-crystal of rosiglitazone with berberine (RB) in high-sugar high-fat diet (HSHFD)-induced diabetic KKAy mice.

**Methods:** Diabetic KKAy mice were randomly divided into seven groups: KKAy model control group (DM control) treated with 3% sodium carboxymethyl cellulose; RB groups, administered daily with RB 0.7 mg/kg (RB-L), 2.11 mg/kg (RB-M), or 6.33 mg/kg (RB-H); positive control groups, administered daily with rosiglitazone 1.04 mg/kg (RSG), berberine 195 mg/kg (BBR), or combination of 1.04 mg/kg RSG and 1.08 mg/kg BBR (MIX). Test compounds were administered orally for 8 weeks. Non-diabetic C57BL/6J mice were used as normal control (NC). Blood glucose, food intake, body weight, glucose-lipid metabolism, and pathological changes in the pancreas and liver were examined. We further evaluated the mechanism of action of RB in C2C12 and HepG2 cells stimulated with high glucose and palmitate.

**Results:** RB treatment improved glucolipid metabolism and insulin resistance in diabetic KKAy mice. RB reduced blood glucose levels, white fat index, plasma triglyceride (TG), low-density lipoprotein (LDL), gastric inhibitory peptide (GIP), and insulin levels, increased the levels of plasma glucagon-like peptide-1 (GLP-1), high-density lipoprotein (HDL), and glycogen content in the liver and muscle; and improved oral glucose tolerance test (OGTT), insulin tolerance test (ITT), and pathological changes in the pancreas and liver of KKAy mice. Moreover, RB upregulated p-PI3K and p-AKT levels and reduced TXNIP expression in KKAy mice and in HepG2 and C2C12 cells.

**Conclusion:** These data indicate that RB ameliorates insulin resistance and metabolic disorders, and the mechanism might be through regulating the PI3K/AKT/TXNIP signaling pathway *.* Thus, the co-crystal drug RB may be considered as a potential antidiabetic agent for future clinical therapy.

## Introduction

Diabetes mellitus is one of the leading causes of death in the world (Vijan, 2015). More than 400 million people worldwide currently suffer from diabetes, and this number is expected to reach 700 million by 2030, accounting for 10.9% of the global population (Wild et al., 2004). Type 2 diabetes mellitus (T2DM) is a systemic chronic metabolic syndrome with insulin resistance (IR) and/or β-cell dysfunction characterized by developing and worsening hyperglycemia (Pearson, 2019). IR is a bottleneck in the treatment of T2DM and is closely related to various metabolic disorders, including obesity, metabolic syndrome, and fatty liver (Eckel et al., 2005). Therefore, restoring insulin sensitivity, improving metabolic homeostasis, and preventing diabetic complications are crucial for ameliorating T2DM.

Currently, the most common diabetes treatment drugs are anti-hyperglycemic agents that reduce the detrimental effects of hyperglycemia (Lancet, 2017). The development of antidiabetic drugs has made significant progress, including insulin and oral hypoglycemic agents such as biguanides, thiazolidinediones, sulfonylureas, and alpha-glucosidase inhibitors. However, deficiencies still exist (Brietzke, 2015). At present, few hypoglycemic drugs can maintain stable blood sugar levels for years (Khunti et al., 2013; Aschner et al., 2020), and their potential toxicity and side effects have attracted increasing attention (Brietzke, 2015).

Metformin is the first-line drug for T2DM treatment. Its discovery is linked to *Ginkgo biloba*, a traditional herbal medicine (Bailey, 2017), prompting research to identify new hypoglycemic agents from traditional Chinese herbs. Berberine (BBR), an isoquinoline alkaloid isolated from *Coptis chinensis* or *Phellodendron Phellodendri*, improves insulin resistance and decreases blood glucose and lipid levels (Pirillo and Catapano, 2015; Xu et al., 2021). Its hypoglycemic effect is nearly similar to metformin (Zhang et al., 2010). Nevertheless, applications of BBR are limited owing to its poor water solubility and low bioavailability (Tan et al., 2011; Li et al., 2018). Rosiglitazone (RSG), a classic insulin sensitizer, improves lipid and glucose metabolism by activating PPAR-γ (Lebovitz, 2019). However, adverse reactions, such as risk of cardiac events and fluid retention, limit its clinical application (Richter et al., 2007). Therefore, development of hypoglycemic drugs with good efficacy, fewer side effects, and lower cost has always been a research hotspot in the medical field.

Pharmaceutical co-crystallization has been used to form various active pharmaceutical ingredients by utilizing the formation of hydrogen or other non-covalent bonds to improve the bioavailability, solubility, and stability of chemicals (Sun, 2013; Rodrigues et al., 2018). Pharmaceutical co-crystallization has received widespread attention in recent years because of its combined therapeutic effects, ability to reduce the dose or number of doses administered, and reduction in adverse effects (Qiao et al., 2011; Srivastava et al., 2018). Several pharmaceutical co-crystal formulations have been developed to treat cardiovascular and metabolic diseases, such as entresto for heart failure (Solomon et al., 2019), pasiniazid as anti-tuberculosis (Zhang Y. et al., 2020; Ying et al., 2021), ertugliflozin and suglat for diabetes (Cannon et al., 2020; Tahara et al., 2012).

RB, a non-covalent adduct co-crystal drug, was synthesized using RSG and BBR in a 1:1 M ratio (Guan et al., 2020). RB is expected to combine the therapeutic advantages of RSG and BBR in improving insulin resistance and complications in patients with T2DM while reducing the dosage or administration frequency and increasing the bioavailability. In this study, we investigated the hypoglycemic effects of RB and the possible mechanisms. Through high-fat and high-sugar diet (HSHFD)-induced T2DM model KKAy mice, we explored the effect of RB on body weight, blood glucose, glucose tolerance, insulin tolerance, insulin resistance index, glucolipid metabolism, and serum GIP and GLP-1 in T2DM mice. We also explored the underlying mechanisms to provide a theoretical basis for therapeutic action of RB in patients with T2DM.

## Materials and Methods

### Chemicals, Reagents, and Antibodies

RB was developed by the School of Pharmacy, Chongqing Medical University. It was synthesized as described (Guan et al., 2020). RSG sodium tablets were purchased from Taiji Group Chongqing Fuling Pharmaceutical Factory Co., Ltd. (Chongqing, China). BBR chloride was purchased from West Plant Extraction Factory (Sichuan, China). Sodium carboxymethyl cellulose (CMC) was purchased from Semick Biotechnology Co. Ltd. (Chongqing, China). Primary antibodies against PI3K (p85) (#4257), phospho-AKT (Ser473) (#4060S), AKT (#4691S), and TXNIP (#14715S) were obtained from Cell Signaling Technology (Danvers, MA, United States). Primary antibody against phosphor-PI3K (#4257) was obtained from Affinity Biosciences LTD. (OH, United States). β-Actin (sc-47778) was purchased from Santa Cruz Biotechnology, Inc. (Dallas, TX, United States). Horseradish peroxidase-conjugated secondary antibodies were purchased from Zhongshan Jinqiao Biotechnology Co. Ltd. (Beijing, China). 2-[N-(7-nitrobenz-2-oxa-1,3-diazol-4-yl) amino]-2-deoxy-d-glucose (2-NBDG) was purchased from Good Laboratory Practice Bioscience Technology (Montclair, CA, United States). RSG, mannitol, PI3K inhibitor LY294002, and palmitic acid were purchased from Medchem Express (NJ, United States). D-(+)-glucose and insulin were purchased from Beyotime Biotechnology (Beijing, China). 3-(4,5-Dimethylthiazol-2-yl)-2,5-diphenyltetrazolium bromide (MTT) was purchased from Sangon Biotech Co., Ltd. (Shanghai, China). The Bio-Plex Pro™ Mouse Diabetes Panel 8-Plex Kit was purchased from Bio-Rad Laboratories (Hercules, CA, United States). Dulbecco’s modified Eagle medium (DMEM) and horse serum (HS) were obtained from Gibco (Thermo Fisher Scientific Inc., Waltham, MA, United States). Fetal bovine serum (FBS) was obtained from Procell Life Science and Technology Co., Ltd. (Wuhan, China). A glycogen detection kit was purchased from Solarbio Science and Technology Co., Ltd. (Beijing, China). The glucose test kit was obtained from the Nanjing Jiancheng Bioengineering Institute (Nanjing, China).

### Animals

Male KKAy mice (4–5 weeks old) and age-matched C57BL/6J mice were purchased from Beijing Huafukang Biology Technology Co. Quality testing of mice was performed by the Experimental Animal Center, Chinese Academy of Medical Science, Beijing, China (Approval number: SCXK Beijing-2019-0008). Unless otherwise stated, all mice were allowed access to food and water *ad libitum*. Specific pathogen-free (SPF) rooms were maintained at 22°C, 50% humidity, and a 12-h light/dark cycle. The experiment was performed according to the principles and guidelines of the Chinese Council for Animal Care and was approved by the Institutional Animal Care and Use Committee of Chongqing Science and Technology Committee.

### Mice Treatment

C57BL/6J mice were fed a standard chow diet, and KKAy mice were fed a high-sugar high-fat diet (HSHFD) during the experiment. All mice were fed adaptively for 2 weeks. T2DM KKAy mice were monitored for blood glucose levels, and mice with levels >11.1 mM were selected randomly for the experiment. The mice were divided into eight groups: C57BL/6J normal control group (NC), diabetes mellitus control group (DM control), RSG group (RSG, 1.04 mg/kg/day), BBR group (BBR, 195 mg/kg/day), RSG (1.04 mg/kg/day) plus BBR (1.08 mg/kg/day) group (MIX), RB groups (0.7 mg/kg/day, RB-L; 2.11 mg/kg/day, RB-M; 6.33 mg/kg/day, RB-H). All drugs were dissolved in 0.3% CMC. Both the NC and DM control groups were treated with 0.3% CMC (0.1 ml/10 g). Medications were administered intragastrically once daily for eight consecutive weeks. Blood glucose levels were measured weekly using a blood glucose meter (ONETOUCH Ultra Lifecan, United States). Bodyweight was monitored weekly. Food intake was monitored every 2 days. All mice were sacrificed by cervical dislocation after 8 weeks of treatment, and the liver, muscle tissue, white adipose tissue, and pancreas were collected.

### Oral Glucose Tolerance Test

Mice were fasted for 12 h, followed by oral glucose administration (2 g/kg body weight, p.o.). Whole venous blood was obtained from the tail vein at 0, 30, 60, 90, and 120 min after administration. Blood glucose levels were measured using an automatic glucometer (ONETOUCH Ultra, Lifecan, United States). The area under the curve (AUC) was calculated by using GraphPad software 8.

### Insulin Tolerance Test

Mice were fasted for 12 h and then injected insulin intraperitoneally (0.75 units/kg body weight, i.p.). Whole venous blood was obtained from the tail vein at 0, 15, 30, 60, 90, and 120 min after administration. Blood glucose levels were measured using an automatic glucometer (ONETOUCH Ultra, Lifecan, United States). The area under the curve (AUC) was calculated by using GraphPad software 8.

### Glucose and Lipid Metabolism Test

Orbital venous plexus blood and white fat were collected from mice after the 8-week treatment; blood serum was separated. Serum triglyceride (TG), high-density lipoprotein (HDL), and low-density lipoprotein (LDL) levels were measured using an auto-biochemical analyzer (Mindray BS-220, China). Fasting blood glucose (FBG) levels were measured using a blood glucose meter. Fasting insulin (FIN), gastric inhibitory peptide (GIP), and glucagon-like peptide-1 (GLP-1) levels were determined using Bio-Plex suspension chip system (Bio-Plex 200 System) with Bio-Plex Pro™ Assay kit, and the insulin resistance index (HOMA-IR) and insulin sensitivity index (ISI) were calculated. Following the manufacturer’s protocol, glycogen content in the liver and muscle was measured using respective assay kits. Similarly, glucose consumption and cellular glycogen content were measured using assay kits. HOMA-IR index = FBG (mM) × FIN (mIU/L)/22.5; ISI = 1/[FBG (mM) × FIN (mIU/L)]; white fat index (WFI) = white fat (mg)/body weight of mouse (g).

### Hematoxylin and Eosin Staining

All mice were sacrificed by cervical dislocation after 8 weeks of treatment, and the liver and pancreas were collected. Mouse tissues were post-fixed in 4% paraformaldehyde for 24 h and sectioned after embedding in paraffin. Sections were prepared and stained with H&E using standard procedure. Slides were examined under Nikon ECLIPSE Ci biological microscope and images were captured using Nikon color digital camera.

### Cell Culture and Stimulation

C2C12 myoblasts and HepG2 cells were cultured in DMEM supplemented with 10% FBS in a 5% CO_2_ incubator at 37°C. For differentiation of C2C12 myoblasts, the cells were cultured in DMEM containing 2% HS for 5 days. C2C12 myotubes and HepG2 cells were transferred to 33.3 mM high-sugar DMEM medium (H-DMEM) containing 0.25 mM palmitate (PA) for 36 h and then treated with the corresponding drugs for 24 h to establish an insulin resistance model. Cells were divided into eight groups: normal control group (NC) treated with the same volume of DMEM, model group (DM) treated with H-DMEM and PA, RSG group treated with H-DMEM and PA + RSG (36 μM), BBR group treated with H-DMEM and PA + BBR (36 μM), MIX group treated with H-DMEM and PA + RSG (36 μM) combined with BBR (36 μM), RB-L group treated with H-DMEM and PA + RB (4 μM), RB-M group treated with H-DMEM and PA + RB (12 μM), and RB-H group treated with H-DMEM and PA + RB (36 μM). In addition, the mannitol group (MAN) was treated with a final concentration of 33.3 mM mannitol for the same duration as the DM control group.

### Glucose Uptake Assay

HepG2 cells were cultured in 24-well plates at a density of 1.5 × 10^4^ cells per well and pretreated as described above. After treatment, the cells were washed twice with PBS and then incubated with 0.1 mM 2-NBDG at 37°C for 30 min. Glucose uptake was terminated by three quick washes with ice-cold PBS and recorded using a fluorescence inversion microscope. The average fluorescence intensity was evaluated using the ImageJ software.

### Western Blotting Analysis

Proteins (30–50 μg) were electrophoresed on 8% SDS-PAGE gels and then transferred to PVDF membranes (Millipore, MA, United States). Next, the membranes were blocked for 2 h at room temperature with 5% non-fat dry milk in Tris-buffered saline with Tween-20 (TBST) and then incubated with primary antibodies at a dilution of 1:1,000 for 12 h at 4°C. After washing off the excess primary antibodies with TBST, the membranes were incubated with the appropriate secondary antibodies at a 1:5,000 dilution for 2 h at room temperature. The membranes were washed thrice with TBST for a total of 30 min. An ECL kit was used to enhance chemiluminescence, and a scanner was used to quantify the protein bands. Signal bands were quantified by densitometric analysis using the ImageJ software.

### Statistical Analysis

All results are expressed as the mean ± SD. Data were examined using one-way analysis of variance (ANOVA) followed by Tukey’s comparisons. Statistical significance was set at *p* < 0.05. GraphPad software 8 (San Diego, CA, United States) was used for calculations.

## Results

### RB Regulates Blood Glucose, Body Weight, and Food Intake in KKAy Mice

**FIGURE 1:**
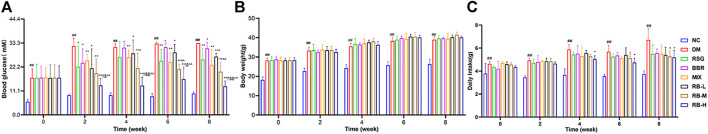
Physiological characteristics of RB-treated diabetic KKAy mice. **(A)** Random blood glucose; **(B)** Body weight; **(C)** Daily food intake. Data are expressed as the mean ± SD, *n* = 7 per group. ^#^ *p* < 0.05, ^##^ *p* < 0.01, vs. NC group; ^*^ *p* < 0.05, ^**^ *p* < 0.01, vs. DM control group; ^$^ *p* < 0.05, ^$$^ *p* < 0.01, vs. RSG group; ^^^ *p* < 0.05, ^^^^ *p* < 0.01, vs. BBR group; ^!^ *p* < 0.05, ^!!^ *p* < 0.01, vs. MIX group.

We conducted *in vivo* experiments using an HSHFD-induced diabetic mouse model KKAy mice to investigate the effects of RB treatment. All selected KKAy mice had blood glucose levels >11.1 mM, which accorded with the characteristics of the diabetic model (Figure 1A). Randomized glucose levels were consistently elevated in the DM control group compared to those in the NC group and peaked at week 6 (>33.3 mM). Compared to the DM control group, the blood glucose levels of the treatment groups were effectively controlled after administration, particularly in the RB-M and RB-H groups. The mean random blood glucose levels in the RB-M group were ameliorated to a greater extent than those in the positive control groups (RSG, BBR, and MIX). However, there were no statistically significant differences among the groups. Additionally, bodyweight of the mice in each group gradually increased throughout the experiment. The weight of mice in the DM control group was always higher than that of mice in the NC group. However, there was little difference between the treatment and DM control groups (Figure 1B). Our results confirmed that the treatment did not affect the body weight of KKAy mice. Moreover, the average food intake in the DM control group was significantly higher than that in the NC group. None of the DM mice showed a difference in food intake until the fourth week. At week 8, the food intake of the RB-M and RB-H groups decreased significantly compared to that of the DM control group (Figure 1C). These data indicated that RB administration could effectively control blood glucose levels in KKAy mice without affecting their body weight.

### RB Improves Glucose Metabolism in HSHFD-Induced Diabetic KKAy Mice

**FIGURE 2:**
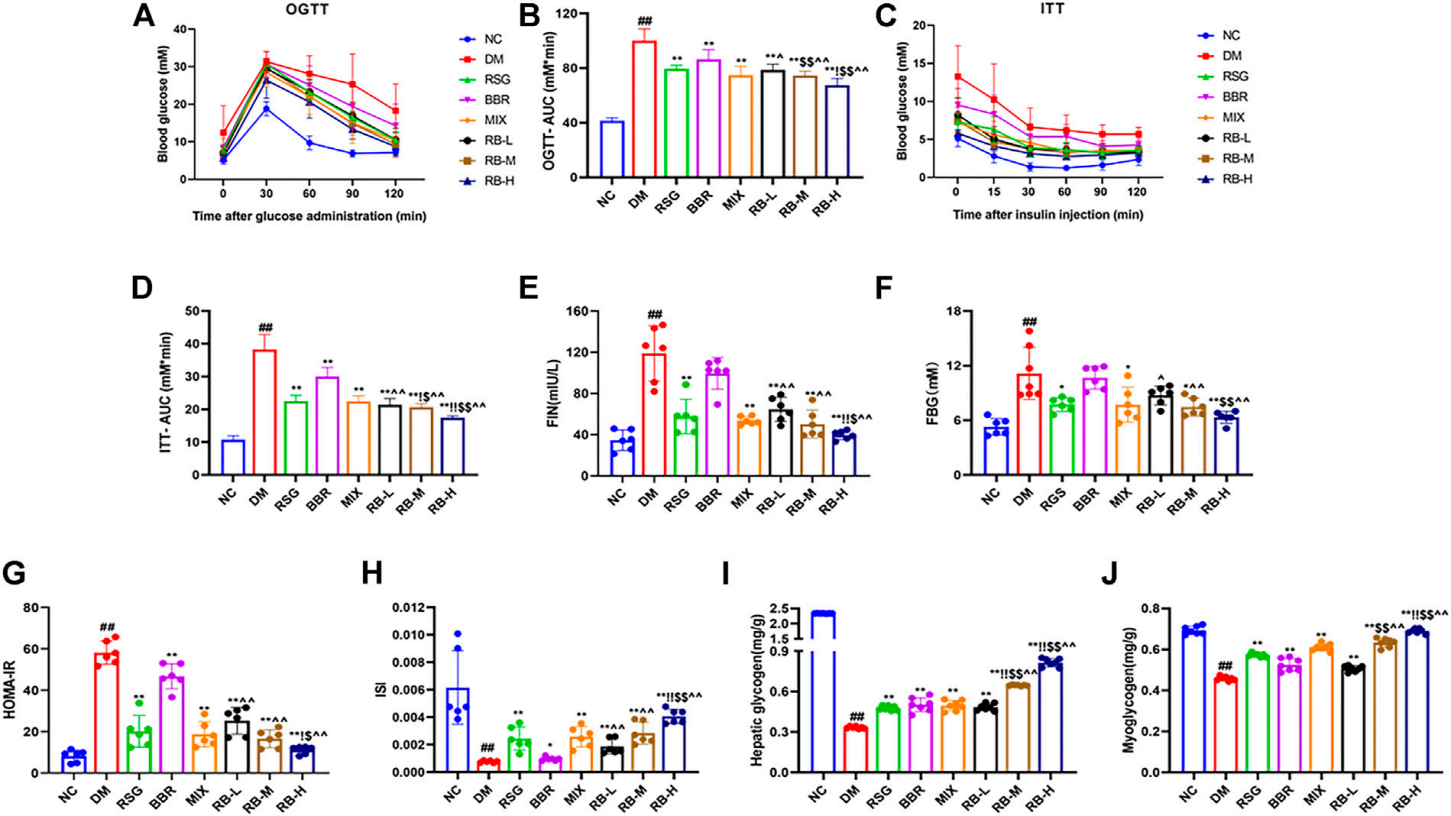
RB improved glucose metabolism in T2DM KKAy mice. **(A)** Oral glucose tolerance test (OGTT), *n* = 7 per group; **(B)** Area under the curve (AUC) of OGTT; **(C)** Insulin tolerance test (ITT), *n* = 7 per group; **(D)** AUC of ITT; **(E)** Fasting serum insulin (FIN); **(F)** Fasting blood glucose (FBG); **(G)** Homeostasis model assessment of insulin resistance (HOMA-IR); **(H)** Insulin sensitivity index (ISI); **(I)** Hepatic glycogen content; **(J)** Myoglycogen content. Data are expressed as the mean ± SD. ^#^ *p* < 0.05, ^##^ *p* < 0.01, vs. NC group; ^*^ *p* < 0.05, ^**^ *p* < 0.01, vs. DM control group; ^$^ *p* < 0.05, ^$$^ *p* < 0.01, vs. RSG group; ^^^ *p* < 0.05, ^^^^ *p* < 0.01, vs. BBR group; ^!^ *p* < 0.05, ^!!^ *p* < 0.01, vs. MIX group.

To determine whether RB affects insulin sensitivity *in vivo*, T2DM KKAy mice were treated with RB for 8 weeks. OGTT testing showed that the blood glucose levels of DM mice increased significantly after glucose administration (0.5 h) and reached a peak. After 120 min, the blood glucose level remained relatively high (Figure 2A). The basal blood glucose level in the RB groups (RB-L, RB-M, and RB-H) was lower than that in the DM control group, and the blood glucose level after glucose administration was elevated. After 30 min of glucose administration, the blood glucose levels in the RB-H group were lower than those in the RB-M and RB-L groups, with all significantly lower than those in the DM control group. The effects in the RB groups were dose-dependent. The AUC of the RB-L group was lower than that of the BBR group. The AUC of the RB-M group was lower than that of the RSG and BBR groups. The AUC of the RB-H group was lower than that of the RSG, BBR, and MIX groups, all of which were lower than that of the DM control group (Figure 2B). These data indicate that RB effectively improved impaired glucose tolerance in diabetic KKAy mice.

ITT was performed in the eighth week for comparison to determine the effect of RB on insulin tolerance (Figures 2C,D). During ITT, an apparent difference was observed in blood glucose levels between NC and diabetic mice at any time point. In the ITT experiment, mice in the NC group showed rapid decrease followed by slow decrease in blood sugar levels compared with KKAy mice, whereas mice in the DM control group exhibited higher blood sugar and insulin insensitivity, which could be largely relieved by RB treatment. The AUC of the RB group was lower than that of the DM control group, and the blood glucose level of the RB-H group was lower than that of the RB-M and RB-L groups, in a dose-dependent manner. The AUC of the RB-M and RB-H groups was lower than that of the RSG, BBR, and MIX groups, all of which were lower than that of the DM control group. These data demonstrated that RB could effectively improve insulin sensitivity in diabetic KKAy mice.

To investigate changes in insulin resistance, we used the homeostasis model assessment of insulin resistance (HOMA-IR) and insulin sensitivity index (ISI) to evaluate the effect of RB on insulin resistance in KKAy mice. The FIN, FBG, and HOMA-IR indices were higher in the DM control group than in the NC group (Figures 2E–G), indicating the presence of significant systemic insulin resistance in diabetic KKAy mice. The FIN and FBG levels *in vivo* were reversed in the treated groups (Figures 2E,F). The results showed that RB significantly reversed insulin resistance in KKAy mice. HOMA-IR value and plasma insulin concentration showed a decreasing trend (Figure 2G). ISI values showed an increasing trend (Figure 2H), with the most significant in the RB-H group. Moreover, the HOMA-IR index of the RB-H group was lower than that of the RSG, BBR, and MIX groups. The ISI index of the RB-H group was higher than that of the RSG, BBR, and MIX groups. The HOMA-IR index of the RB-M group was lower than that of the RSG, BBR, and MIX groups. The ISI index of the RB-M group was higher than that of the RSG, BBR, and MIX groups. However, the results were statistically different only when compared with the BBR group. These data indicated that RB treatment effectively ameliorated insulin resistance in diabetic KKAy mice.

When the body is insulin-resistant, the liver excessively releases glucose into the bloodstream due to increased glycogenolysis and gluconeogenesis, causing peripheral tissues, such as muscles, to become insensitive to insulin-regulated glucose metabolism. Eventually, reducing glycogen production and blood glucose consumption lead to hyperglycemia (Petersen et al., 2017; Ormazabal et al., 2018). Our data showed that KKAy mice had significant insulin resistance and low levels of hepatic glycogen and myoglycogen compared with the NC group. After RB treatment, the liver glycogen and myoglycogen contents of KKAy mice significantly increased (Figures 2I,J). Furthermore, RB increased glycogen stores in the liver and muscle in a dose-dependent manner, evidenced by higher muscle glycogen content in the RB-M and RB-H groups than in the RSG and BBR groups and higher liver glycogen content in the RB-M and RB-H groups than in the RSG, BBR, and MIX groups. The data indicate that RB effectively improved glucose utilization and metabolism in diabetic KKAy mice.

### RB Modulates Lipid Metabolism in HSHFD-Induced Diabetic KKAy Mice

**FIGURE 3:**
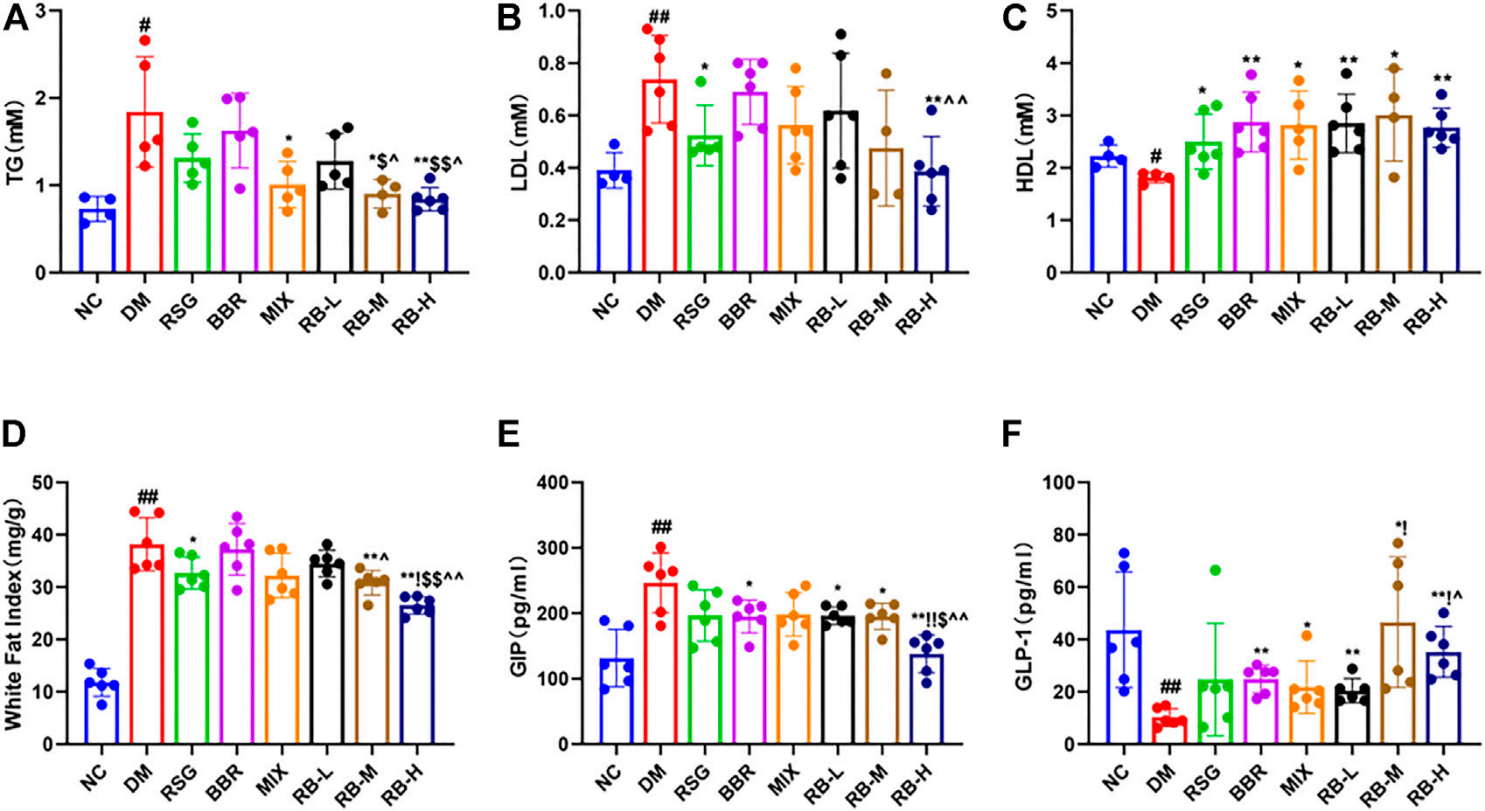
RB ameliorated lipid metabolism in diabetic KKAy mice. **(A)** Triglyceride (TG); **(B)** Low-density lipoprotein (LDL); **(C)** High-density lipoprotein (HDL), inconsistent numbers of mice detected in A,B, and C plots due to insufficient serum samples; **(D)** White fat index (WFI); **(E)** Gastric inhibitory polypeptide (GIP); **(F)** Glucagon-like peptide-1 (GLP-1). Data are expressed as the mean ± SD. ^#^ *p* < 0.05, ^##^ *p* < 0.01, vs. NC group; ^*^ *p* < 0.05, ^**^ *p* < 0.01, vs. DM control group; ^$^ *p* < 0.05, ^$$^ *p* < 0.01, vs. RSG group; ^^^ *p* < 0.05, ^^^^ *p* < 0.01, vs. BBR group; ^!^ *p* < 0.05, ^!!^ *p* < 0.01, vs. MIX group.

Compared with the NC group, significant changes in lipid levels were observed in the DM control group, including elevated TG and LDL levels and decreased HDL in the serum of KKAy mice (Figures 3A–C). After 8 weeks of RB treatment, the serum TG concentration in the RB-M and RB-H groups was lower than that in the positive control groups (RSG, BBR, and MIX). However, the differences were statistically significant only when compared with the RSG and BBR groups (Figure 3A). The serum LDL concentration in the RB-M and RB-H groups was lower than that in the positive control groups and significantly different between the RB-H and BBR groups (Figure 3B). The serum HDL concentration in the RB-M group was higher than that in the positive control groups, but the difference was not statistically significant (Figure 3C). The WFI in the RB-M and RB-H groups was lower than that in the positive control groups; differences were statistically significant between the RB-M group and BBR groups and the RB-H and positive control groups (Figure 3D). These findings suggest that RB administration ameliorates dyslipidemia in T2DM mice, contributing to the improvement of insulin resistance and playing an important role in antidiabetic therapies.

The hormones GIP and GLP-1 are secreted by the gut. GLP-1 is a multifaceted hormone with broad pharmacological potential. GLP-1 receptor agonists have been successfully used clinically for T2DM treatment, whereas GIP levels are increased in individuals with obesity and T2DM (Tasyurek et al., 2014; Holst and Rosenkilde, 2020). The GIP levels in the DM control group were significantly higher than those in the NC group (Figure 3E). The medication intervention significantly decreased the GIP level, particularly in the RB-H group that had GIP level lower than that of the positive control groups (*p* < 0.01) and close to that of the NC group. Meanwhile, slight difference was observed between the RB-L and RB-M groups and the positive control groups. Additionally, GLP-1 (Figure 3F) in the DM control group was reduced by three-fold compared to that in the NC group. GLP-1 levels tended to increase in response to medical stimulation. Notably, the increase in GLP-1 levels was concentration-relevant rather than concentration-dependent with RB treatment. The RB-M group showed increased level of GLP-1 compared with the NC group in T2DM KKAy mice, and the GLP-1 level in the RB-M and RB-H groups was higher than that in the positive control groups but statistically significant only when compared with the BBR group. These data suggest that RB may improve insulin resistance by increasing serum GLP-1 concentration and decreasing GIP concentration, thereby regulating lipid metabolism, reducing appetite, and protecting islet cells.

### RB Ameliorates Liver and Pancreatic Lesions in Diabetic KKAy Mice

**FIGURE 4:**
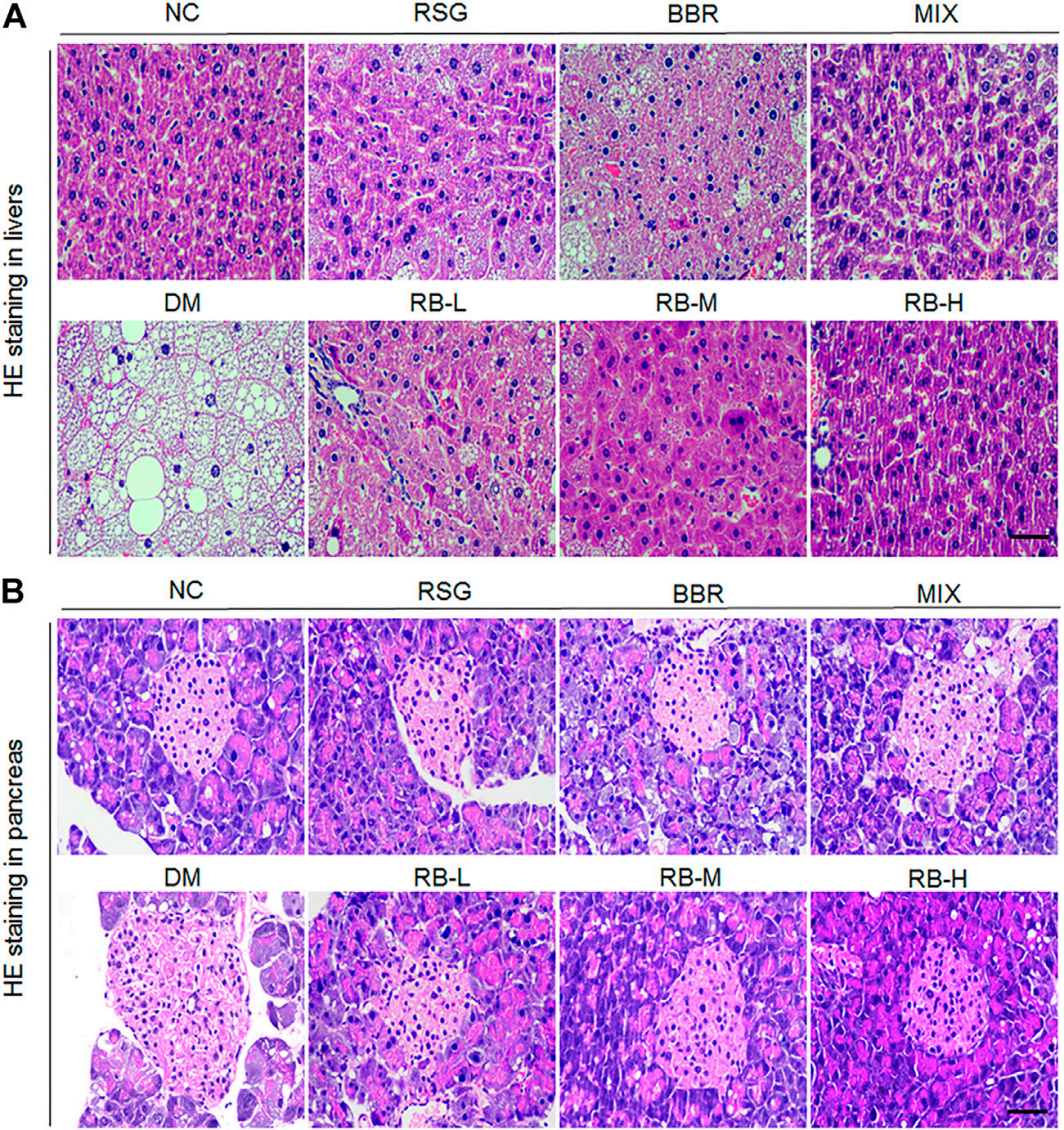
Effects of RB on histopathological alterations in KKAy mice. **(A)** Hepatic histopathological alterations; **(B)** Pancreatic islet histopathological alterations. Scale bar, 50 μm.

We evaluated whether RB treatment had any effect on T2DM-induced hepatocyte alterations and pancreatic islet cells through examining histopathological changes using H&E staining in T2DM KKAy mice after RB intervention. We observed that the hepatocytes were arranged neatly, with no steatosis observed in the NC group (Figure 4A). In contrast, swollen hepatocytes and severe fatty degeneration were observed in the liver cells, with a massive accumulation of fat vesicles in the cytoplasm in the DM control group. Ameliorative effect of steatosis in the RSG and MIX groups was improved compared with that in the DM control group. However, this improvement was not significant in the BBR group. RB treatment was significantly superior to the positive control groups (RSG, BBR, MIX) and reduced liver lipid accumulation in KKAy mice in a dose-dependent manner. Similarly, the pancreatic structure was clear and complete in the NC group, and slightly stained islet cells were arranged in groups (Figure 4B). The β cells were close to each other without obvious cell degeneration or necrosis. In the DM control group, islet cells were hypertrophic and disorganized, with swollen, deformed, and loosely arranged islet β cells. The degree of morphological and structural damage to islet cells was significantly lower in the RB administered groups than in the DM control group. RB treatment significantly repaired pancreatic islet cell damage in a concentration-dependent manner, consistent with hepatic alterations.

### RB Improves Glucose Metabolism *In Vitro*

**FIGURE 5:**
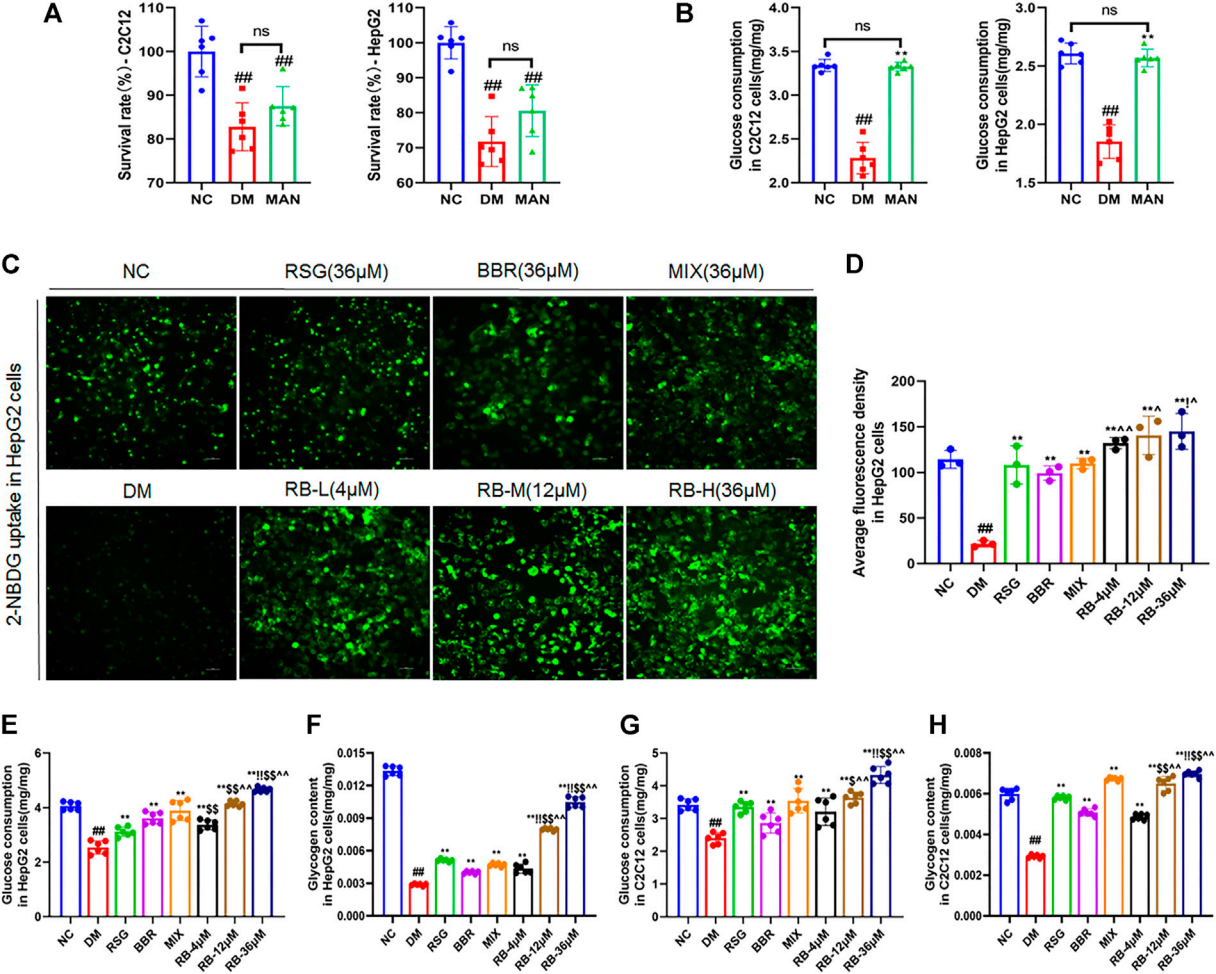
RB regulated glucose metabolism *in vitro*. **(A,B)** Effect of mannitol (MAN) on the survival rate and glucose consumption in C2C12 and HepG2 cells; **(C)** RB restores 2-NBDG uptake in insulin-resistant HepG2 cells; **(D)** Average fluorescence intensity of 2-NBDG in HepG2 cells; **(E)** Glucose consumption in HepG2 cells; **(F)** Glycogen content in HepG2 cells; **(G)** Glucose consumption in C2C12 cells; **(H)** Glycogen content in C2C12 cells. Data are expressed as the mean ± SD. ^#^ *p* < 0.05, ^##^ *p* < 0.01, vs. NC group; ^*^ *p* < 0.05, ^**^ *p* < 0.01, vs. DM control group; ^$^ *p* < 0.05, ^$$^ *p* < 0.01, vs. RSG group; ^^^ *p* < 0.05, ^^^^ *p* < 0.01, vs. BBR group; ^!^ *p* < 0.05, ^!!^ *p* < 0.01, vs. MIX group; ns, no significance.

To examine the role of RB in regulating glucose metabolism *in vitro*, high glucose (33.3 mM) and palmitate were used to establish a cellular model of insulin resistance, and the glucose analog 2-NBDG was used as a radiotracer to track glucose uptake. First, to confirm the experimental role of osmolarity induced by high glucose, mannitol (MAN), corresponding to high glucose concentration in the DM control group, was used to assess the effect of a high osmotic environment. The data showed that both the high glucose and mannitol groups (MAN) had decreased cell survival rates in HepG2 and C2C12 cells compared to the normal control group (NC), and there was no statistically significant difference between the MAN and DM control groups (Figure 5A). Moreover, the addition of high glucose significantly reduced glucose uptake, whereas MAN did not alter glucose consumption in HepG2 and C2C12 cells compared to the NC group (Figure 5B). In short, although osmolarity induced by high glucose and the same concentration of mannitol affected cell viability, mannitol had no significant effect on glucose uptake, excluding the effect of a hyperosmotic environment on glucose uptake in HepG2 and C2C12 cells.

Next, we examined whether RB affected glucose uptake in insulin-resistant HepG2 cells (DM) (Figures 5C,D). Insulin resistance in hepatocytes causes impaired insulin signaling and decreased glucose uptake, the major contributors of hyperglycemia. Treatment of HepG2 cells with high glucose and palmitate (PA) triggers a significant reduction in insulin signaling leading to insulin resistance (Wen et al., 2011; Palomer et al., 2018). Our results showed that glucose uptake was significantly lower in DM cells than in NC cells. After treatment with RB (RB-L, RB-M, and RB-H), glucose uptake remarkably increased in a concentration-dependent manner compared to DM cells. Similarly, treatment with 36 μM RSG, BBR, and MIX increased glucose uptake compared to DM cells. The RB-H group showed better glucose uptake than the positive control groups (RSG, BBR, and MIX) at the same concentration. Our data demonstrated that insulin-resistant cells had significantly impaired glucose uptake and that the use of RB increased sugar uptake and further decreased insulin resistance.

Similarly, corresponding effects were observed for cell glucose consumption and glycogen storage. In HepG2 and C2C12 cells, glucose consumption and glycogen stores were significantly reduced in the DM control group compared to the NC group and reversed with RB treatment (Figures 5E–H). Moreover, RB improved cellular glucose consumption and glycogen storage in a dose-dependent manner, which was superior to that of the RSG, BBR, and MIX groups at the same dose (36 μM). Our data suggest that RB effectively restores glucose consumption and glycogen stores and regulates glucose metabolism in insulin-resistant HepG2 and C2C12 cells.

### RB Ameliorates Insulin Resistance Through the PI3K/AKT/TXNIP Cascade Signaling Pathway

**FIGURE 6:**
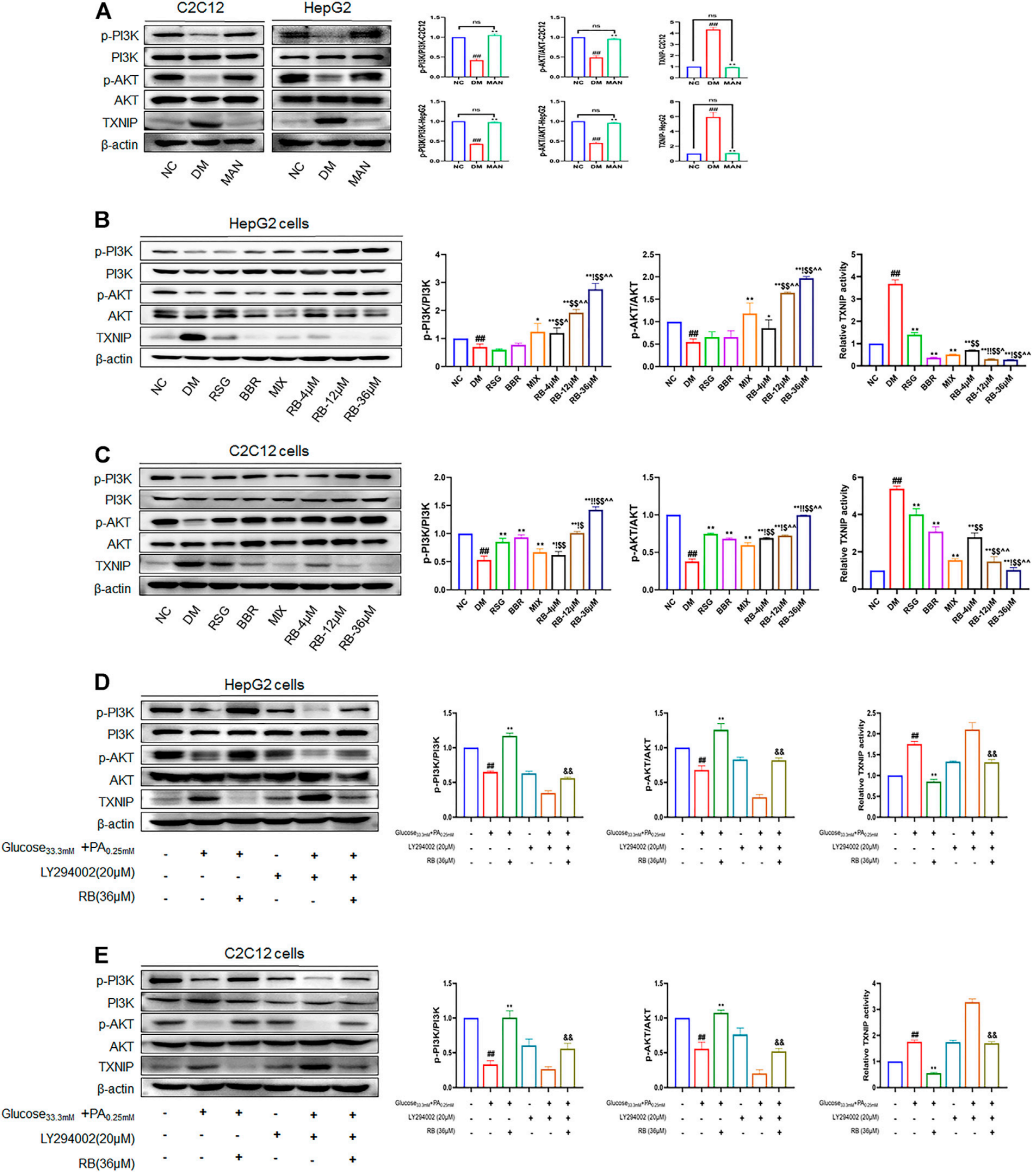
RB ameliorated insulin resistance by upregulating the PI3K/AKT/TXNIP cascade signaling pathway *in vitro*. **(A)** Effect of MAN on the expression of p-PI3K, PI3K, p-AKT, AKT, and TXNIP proteins in C2C12 and HepG2 cells; **(B)** Effect of RB on the expression of p-PI3K, PI3K, p-AKT, AKT, and TXNIP proteins in HepG2 cells; **(C)** Effect of RB on the expression of p-PI3K, PI3K, p-AKT, AKT, and TXNIP proteins in C2C12 cells; **(D)** Effect of PI3K inhibitor LY294002 (20 μM) on the expression of p-PI3K, PI3K, p-AKT, AKT, and TXNIP proteins in HepG2 cells; **(E)** Effect of PI3K inhibitor LY294002 (20 μM) on the expression of p-PI3K, PI3K, p-AKT, AKT, and TXNIP proteins in C2C12 cells. All data are expressed as the mean ± SD of three independent experiments. ^#^ *p* < 0.05, ^##^ *p* < 0.01, vs. NC group; ^*^ *p* < 0.05, ^**^ *p* < 0.01, vs. DM control group; ^$^ *p* < 0.05, ^$$^ *p* < 0.01, vs. RSG group; ^^^ *p* < 0.05, ^^^^ *p* < 0.01, vs. BBR group; ^!^ *p* < 0.05, ^!!^ *p* < 0.01, vs. MIX group; ^&^ *p* < 0.05, ^&&^ *p* < 0.01, vs. RB group; ns, no significance.

To further test the ability of RB to modulate the insulin signaling pathway, PI3K, AKT, and TXNIP were used as critical messengers. Insulin activates the PI3K/AKT pathway by binding to the insulin receptor that regulates glucose and lipid metabolism (Holland et al., 2007; Saltiel, 2021). Damage to the PI3K/AKT pathway leads to the development of insulin resistance and T2DM (Huang et al., 2018). As an important regulator of cell metabolism and stress, TXNIP, mediated by AKT under glucose stress, is often upregulated in T2DM (Robinson et al., 2013; Hong et al., 2016; Waldhart et al., 2017). To investigate the expression levels of these proteins and confirm the results of the above experiments, western blot analysis was performed. First, high glucose caused noticeably lower PI3K and AKT phosphorylation levels in HepG2 and C2C12 cells (DM control group) and significantly higher TXINP protein levels than that of the NC group (Figure 6), indicating the successful establishment of an insulin-resistant cell model. However, mannitol stimulation with high glucose isotonicity in the DM control group did not alter the expression of p-AKT, p-PI3K, or TXNIP, showing little effect of mannitol on PI3K/AKT/TXNIP signaling regulation (Figure 6A). As shown in Figure 6B, RB treatment increased the expression of p-AKT and p-PI3K. It decreased the expression of TXNIP in a dose-dependent manner in HepG2 cells, with the maximal effect observed at a concentration of 36 μM, superior to that of the RSG, BBR, and MIX groups at the same concentration. The same verification was performed for C2C12 cells. We found a significant increase in p-PI3K and p-AKT expression with RB concentration, superior to RSG, BBR, and MIX groups (Figure 6C). This indicated that RB could activate the PI3K/AKT pathway inhibited by high glucose and palmitate, and mediate the downregulation of TXNIP, consistent with previous studies (Yoshihara et al., 2010; Hong et al., 2016). We then used a specific PI3K inhibitor, LY294002, to investigate whether RB ameliorates insulin resistance through the PI3K/AKT/TXNIP pathway *in vitro* (Figures 6D,E). HepG2 and C2C12 cells were pretreated with LY294002 (20 μM) for 2 h, followed by treatment with 36 μM RB for 24 h. As shown in Figures 6D and E, the phosphorylation levels of PI3K and AKT were significantly inhibited by LY294002 pretreatment and high glucose + PA stimulation in HepG2 and C2C12 cells. Moreover, high glucose + PA stimulation elevated TXNIP protein expression levels, enhanced after LY294002 pretreatment. In addition, the phosphorylation of PI3K and AKT inhibited by high glucose + PA stimulation was restored and upregulated following treatment with RB. However, LY294002 markedly inhibited RB from restoring PI3K and AKT phosphorylation levels as well as TXNIP levels. As expected, these data confirmed that stimulation with LY294002 significantly attenuated the protective effect of RB on PI3K/AKT/TXNIP signaling.

**FIGURE 7:**
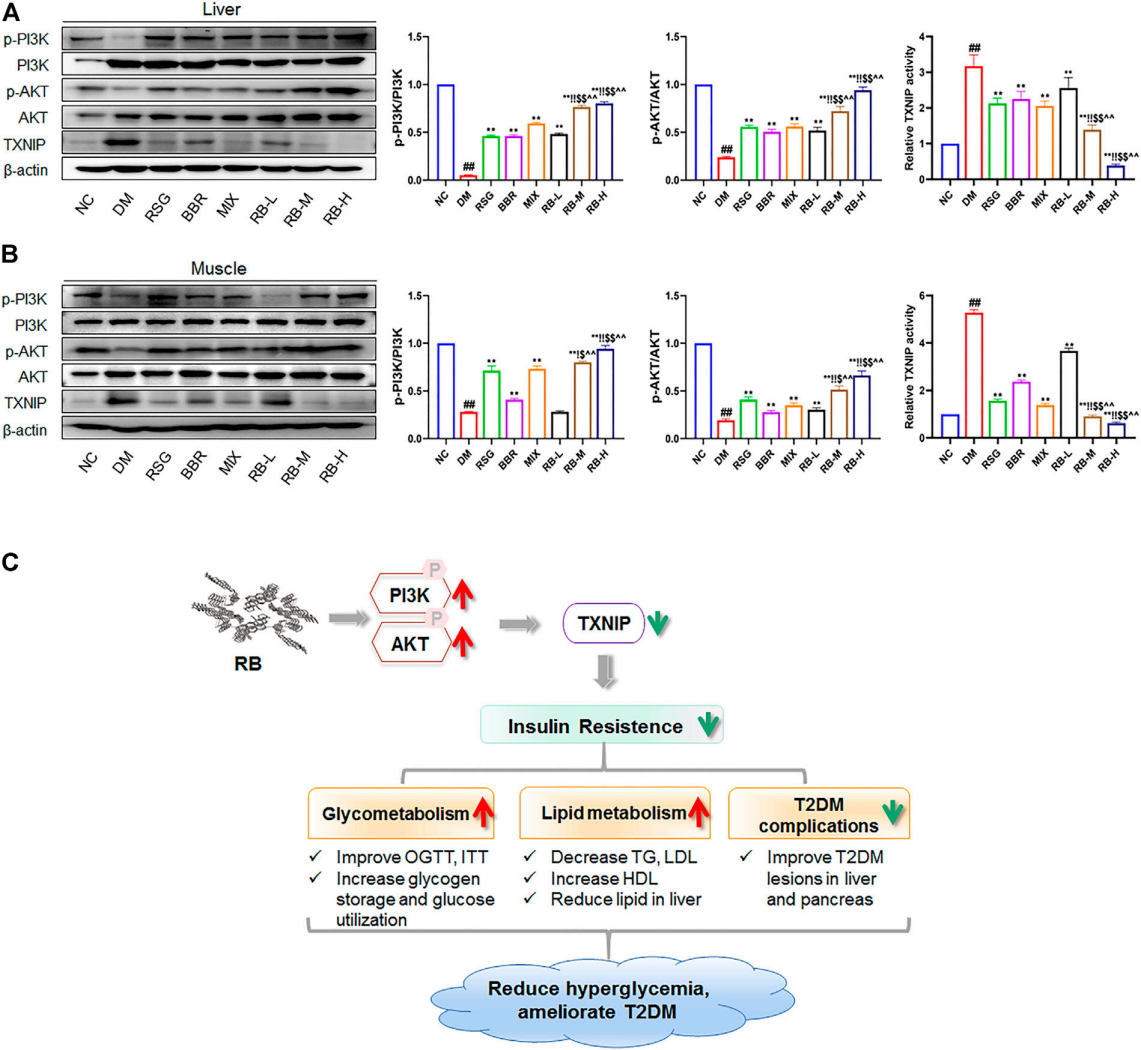
RB ameliorated insulin resistance by upregulating the PI3K/AKT/TXNIP cascade signaling pathway *in vivo*. **(A)** Effect of RB on the expression of p-PI3K, PI3K, p-AKT, AKT, and TXNIP proteins in the liver of KKAy mice; **(B)** Effect of RB on the expression of p-PI3K, PI3K, p-AKT, AKT, and TXNIP proteins in the muscle of KKAy mice. **(C)** Schematic map for RB improving insulin resistance and T2DM. All data are represented as the mean ± SD of three independent experiments. ^#^ *p* < 0.05, ^##^ *p* < 0.01, vs. NC group; ^*^ *p* < 0.05, ^**^ *p* < 0.01, vs. DM control group; ^$^ *p* < 0.05, ^$$^ *p* < 0.01, vs. RSG group; ^^^ *p* < 0.05, ^^^^ *p* < 0.01, vs. BBR group; ^!^ *p* < 0.05, ^!!^ *p* < 0.01, vs. MIX group.

Next, we assessed the action of RB on PI3K/AKT/TXNIP signaling *in vivo*. The phosphorylation and total expression levels of PI3K, AKT, and TXNIP were investigated by protein blotting. The data showed that p-PI3K and p-AKT were downregulated, and that TXNIP protein expression was increased in both the liver and muscle of KKAy mice compared to C57BL/6J mice (NC group) (Figures 7A,B). RB treatment restored the levels of p-PI3K and p-AKT in the liver and muscle tissues of KKAy mice. Moreover, RB inhibited TXNIP expression in a concentration-dependent manner and was more effective at higher doses. These results further support that RB treatment may ameliorate peripheral diabetic tissue lesions and insulin resistance *via* the PI3K/AKT/TXNIP signaling pathway in KKAy diabetic mice.

## Discussion

The major feature of T2DM is insulin resistance, often accompanied by impaired glucose tolerance (DeFronzo et al., 2015). BBR has been shown to reduce hyperglycemia and inflammation, regulate lipid metabolism and intestinal microorganisms, and improve insulin resistance, diabetic nephropathy, and cardiomyopathy (Pirillo and Catapano, 2015; Xu et al., 2021). BBR may regulate glucose metabolism through multiple mechanisms and signaling pathways, and the hypoglycemic efficacy of BBR is similar to that of metformin (Yin et al., 2008); however, its use is limited by its poor solubility and low bioavailability. RSG, a classic insulin sensitizer can reduce liver glucose output and improve lipid metabolism, glucose utilization, and peripheral tissue sensitivity to insulin by activating PPAR-γ (Richter et al., 2007; Lebovitz, 2019), but its adverse reactions, such as cardiac reactions and fluid retention, limit its clinical application (Brietzke, 2015). A study has shown that compared with oral hypoglycemic drugs alone, such as rosiglitazone or metformin, co-interventions with berberine showed better blood glucose control (Dong et al., 2012). However, the mechanism of action remains unclear. Pharmaceutical co-crystallized RB is synthesized from RSG and BBR in a 1:1 M ratio to improve the stability, dissolution, and bioavailability of the constituents (Guan et al., 2020). We administered the clinically recommended maximum daily dose of RSG to mice as the standard. We then converted it to a median dose group (RB-M, 2.11 mg/kg) based on the ratio of RSG to RB relative molecular mass (357.4/754.81) for the mouse-based model. The dose of RB in low-dose group (RB-L, 0.7 mg/kg) and high-dose (RB-H, 6.33 mg/kg) group were 1/3 and 3 times of the RB-M, respectively. In the current study, we tested whether RB could exert a hypoglycemic effect superior to that of BBR and RSG, alone and in combination, using KKAy mice as model animals. Our study showed that RB ameliorated impaired insulin tolerance (ITT) and glucose tolerance (OGTT). The effect was better compared to the RSG and BBR groups. The improvement of ITT was better than that of the mixture of RSG and BBR. In addition, RB significantly decreased HOMA-IR, increased ISI, and improved systemic insulin sensitivity in KKAy mice.

In T2DM, dyslipidemia is often closely related to insulin resistance characterized by an increase in TC, TG, and LDL and a decrease in HDL (Eckel et al., 2005; Ormazabal et al., 2018). KKAy mice, a spontaneous ideal T2DM mouse model with a metabolic syndrome of hyperglycemia, obesity, and high insulin resistance are widely used in T2DM research since the T2DM development process in KKAy mice is similar to that in humans. Our results showed that RB not only decreased white fat index and the levels of TG and LDL in the peripheral circulation but also increased HDL levels in KKAy mice. Similar results have been previously reported for RSG (Gong et al., 2017). The efficacy of RB was better than that of RSG, BBR, or their mixture to some degree, and the effect of 6.33 mg/kg RB (RB-H) was the best. This is probably due to the formation of co-crystals that improve the drugs’ physical and chemical properties and bioavailability. Amelioration of lipid metabolism in KKAy mice by RB may be related to the enhancement of insulin sensitivity.

GLP-1 and GIP are incretins that play important role in regulating blood glucose and reducing complications in patients with diabetes (Campbell and Drucker, 2013; Nauck and Meier, 2016). Our results show that RB can increase the concentration of GLP-1 and decrease the concentration of GIP in the serum better than that of RSG, BBR, and their mixture. This may be attributed to the increase in BBR dissolution in RB that can improve the intestinal flora (Zhang Z. et al., 2020).

Diabetic liver injury is a common complication of diabetes, with over 50% of T2MD patients suffering from nonalcoholic fatty liver disease (Tilg et al., 2017; Younossi et al., 2019). The fat level in the liver is closely related to insulin resistance, and deterioration of insulin resistance causes islet β cells to secrete excessive insulin. Still, it cannot cater to the need to reduce hyperglycemia, which leads to a compensatory increase in islets and even dysfunction (Eckel et al., 2005; Ježek et al., 2018; Hudish et al., 2019; Tanase et al., 2020). Our results suggested that RB could restore glucose uptake in insulin-resistant hepatocytes HepG2 and improved glycogen content and glucose consumption. In addition, RB reduced hepatic steatosis and improved glucose and lipid metabolism in the liver. These findings were consistent with previous studies (Wei et al., 2019; Zhu et al., 2019), indicating that RB effectively prevents HSHFD-induced liver damage. Our study also showed that RB could improve pancreatic pathological changes and islet compensatory enlargement in KKAy mice. This may be associated with improving systemic insulin resistance and glucolipid metabolism.

Impaired insulin signal transduction and abnormal metabolic pathways are common in T2DM (Saltiel, 2021). The PI3K/AKT signaling pathway is closely linked to the occurrence of diabetes and a decrease in insulin sensitivity; the activation of the PI3K/AKT pathway is blunted with the occurrence of insulin resistance (Huang et al., 2018). Insulin promotes glucose uptake through signal transduction pathways, initiated by binding to the insulin receptor and activating IRS-1 phosphorylation (Kahn, 1985). PI3K regulates glucose metabolism by phosphorylating AKT, which then activates downstream molecules related to insulin signal transduction (Petersen and Shulman, 2018). TXNIP, an important AKT-mediated regulator under glucose stress (Waldhart et al., 2017), is essential for improving glucose and lipid metabolism by regulating β-cell function, liver glucose production, peripheral glucose uptake, and lipogenesis. Overexpression of TXNIP can induce pancreatic β-cell apoptosis, reduce the sensitivity of peripheral tissues to insulin, and decrease energy consumption (Thielen and Shalev, 2018; Yoshihara, 2020). PI3K/AKT pathway promotes glucose uptake and utilization by downregulating TXNIP expression (Hong et al., 2016). In contrast, animals with TXNIP deficiency are immune to diet-induced insulin resistance and T2DM (Yoshihara et al., 2010). Our results showed that RB effectively promoted the uptake and utilization of glucose, increased the phosphorylation of PI3K and AKT, and reduced TXNIP expression in HepG2 and C2C12 cells. The PI3K inhibitor LY294002 could counteract these effects. Moreover, the efficacy of RB was superior to that of RSG, BBR, or their combination. The inhibitory effect of RB on TXNIP may make it a good candidate for the treatment of T2DM.

The efficacy of RB in T2DM was superior to that of RSG, BBR, or their combination treatments. RB alleviates T2DM lesions while improving insulin resistance and is beneficial for mitigating the development and progression of T2DM. However, there are some limitations to our study. Studies of RB toxicity *in vivo* are incomplete, and research on the hypoglycemic mechanism of RB is superficial. The *in vivo* hypoglycemic effect of the BBR positive control is not ideal, consistent with previous reports (Li et al., 2018; Mirhadi et al., 2018), it has poor solubility and bioavailability. On the other hand, it has also been proven that improving the physical and chemical properties of the constituent drugs that benefit from RB co-crystals effectively promotes the efficacy of the drugs.

In conclusion, our study shows that RB improves insulin resistance *in vivo* and *in vitro*, and the mechanism migh be through upregulating the PI3K/AKT signaling therefore suppressing TXNIP expression. RB also ameliorates glucose and lipid metabolism and alleviates diabetes-induced histopathological alterations in the liver and pancreas. These effects are beneficial for treating T2DM and preventing its complications (Figure 7C). Accordingly, RB is expected to combine the therapeutic advantages of RSG and BBR in improving insulin resistance and complications in patients with T2DM while reducing the dosage or administration times and increasing the bioavailability of the drugs.

## Data Availability

The original contributions presented in the study are included in the article/Supplementary Material, further inquiries can be directed to the corresponding authors.

## Glossary

T2DM: Type 2 diabetes mellitus
DM: Diabetes model control
NC: Normal control
RSG: Rosiglitazone
BBR: Berberine
MIX: Combination of RSG and BBR
RB: Co-crystal of rosiglitazone with berberine
RB-L: RB low-dose group
RB-M: RB medium-dose group
RB-H: RB high-dose group
IR: Insulin resistance
OGTT: Oral glucose tolerance test
ITT: Insulin tolerance test
AUC: Area under the curve
CMC: Carboxymethyl cellulose
TG: Triglyceride
HDL: High-density lipoprotein
LDL: Low-density lipoprotein
FBG: Fasting blood glucose
FIN: Fasting insulin
GIP: Gastric inhibitory peptide
GLP-1: Glucagon-like peptide-1
HOMA-IR: Insulin resistance index
ISI: Insulin sensitivity index
WFI: White fat index
PA: Palmitate
H-DMEM: High-sugar DMEM medium
2-NBDG: 2-[N-(7-nitrobenz-2-oxa-1,3-diazol-4-yl) amino]-2-deoxy-d-glucose
TXNIP: Thioredoxin-interacting protein
HSHFD: High-sugar and high-fat diet
IRS: Insulin receptor substrate
PI3K: Phosphoinositide 3-kinase
AKT: Protein kinase B
PVDF: Polyvinylidene fluoride
TBST: Tris-buffered saline with Tween-20
